# Case Report: Galvanic Vestibular Stimulation in the Chronic Spinal Cord Injury Patient

**DOI:** 10.3389/fresc.2022.779846

**Published:** 2022-04-06

**Authors:** Tais Nunes Nascimento, Catarina Costa Boffino

**Affiliations:** Universidade Metodista de São Paulo, São Bernardo do Campo, Brazil

**Keywords:** galvanic vestibular stimulation, spinal cord injury, case report, rehabilitation, non-invasive neuromodulation

## Abstract

The traumatic spinal cord injury can generate sequels with high clinical severity and dysfunction and limitations of irreversible character. Current studies seek to reverse the sequelae and gain functionality in these individuals. Galvanic vestibular stimulation (GVS) has shown to be beneficial in spinal cord function as an evaluation correlated to functionality and for stimulation with physiological and functional characteristics in disease and healthy people. The present study observed the effects of Noise Galvanic Vestibular Stimulation in a patient with chronic spinal cord injury with tetraplegia on postural and trunk control. The evaluations were the Functional Independence Measure (FIM), the American Spinal Injury Association (ASIA) evaluation, and the Clinical Posturography, using force platform to assess postural balance, in the sitting position, through Sensory Organization and Functional Reach Tests. Ten sessions of Noise Galvanic Vestibular Stimulation associated with customized vestibular and neurofunctional rehabilitation were performed. The effects observed were increments in all assessments and tests that include modifications in functional independence, motor and sensory levels, change in disability grade from A (complete) to C (incomplete), and improvements in postural balance and trunk control. The phenomenon of stochastic resonance has shown benefits in postural control in people without vestibular lesions and we could observe some of these phenomena in our patients. We emphasize the need for evaluation with larger populations to observe the phenomena and effects in this group of patients and potential benefits and limitations.

## Introduction

Postural control has been comprehensively studied in the last decades to investigate the systems that are involved in this detailed process. Postural control is the product of the interaction of the individual, task, environment, and context, leaving aside the view of a simple reactive response to a sensory stimulus (1).

Three important systems, visual, somatosensory, and vestibular, which separately provide information on gravity and the surrounding environment, enable the CNS to produce postures that, even in discrete disturbances or demands, trigger compensatory and anticipatory adjustments to maintain balance (2, 3).

The vestibular apparatus's main function is in responding to the acceleration of gravity and linear accelerations of body displacement. It is innervated by the vestibulocochlear nerve, which conducts information up to the central nervous system to the vestibulospinal tracts and the tectospinal tract that control the posture of the head and neck, trunk, and the antigravitational muscles (3–5).

In patients with spinal cord injury, the automatism of balance maintenance is affected; because of the interruption of central communication, patients adopt new patterns of postural control that involve uninvolved parts of the sensory-motor system; the rehabilitation process must stimulate these integral systems to obtain an efficient postural control (6, 7).

The stimulation of whole systems suggests a potential for the rehabilitation of the individual; studies have shown that non-invasive brain stimulation (NIBS) can induce neuroplasticity and a long stimulation can modulate synaptic excitability (8).

A study using galvanic vestibular stimulation in elderly patients was carried out to verify the improvement in stability and postural control; an improvement in the patients' stability, which lasted for hours after the end of the applied stimulus, was seen, suggesting vestibular neuroplasticity (9).

The spinal cord is an important region in our organism, it is a linear, continuous, flexible, and firm structure, with an integral presentation (10–17). Galvanic vestibular stimulation (GVS) generates activation in general, facilitation of muscles, such as those represented in the gait center in the lumbar spinal cord (18). The activation of both the vestibulospinal and reticulospinal pathways saves functional and postural fine-tuning, regulates movement, and is capable of multisegmental stimulation in the spinal cord, which affects multisegmental interneurons regulating motoneuron excitability (18). It seems to activate the neuronal network of the propriospinal system, which is important in neuroplasticity after spinal cord injury, and may even reach segments below the injury, especially in structurally incomplete injuries (18).

Galvanic vestibular stimulation induces modulation in reactions and neural function of long latencies (18) in healthy individuals, and short and long latency reactions were shown to be performed in patients with alteration of the spinal cord by the human T-cell lymphotropic virus type 1 (HTLV-1) virus (19).

It is distinguished from corticospinal activity; this distinction provides an opportunity for quantitative assessment and comparison of residual supraspinal connectivity with the lumbosacral sensorimotor network following the disease and/or spinal cord lesion and especially defines clinically whether the lesion is complete or incomplete (10, 17–20).

In recent studies, implicating specific pathways in mediating adaptive plasticity resulting from a restoration of downward input to sub-injured networks in multiple mammalian models of spinal cord injury that include non-human primates, discriminating between these anatomically and functionally distinct pathways leads to potential as biomarkers for potential recovery after human spinal cord injury (18).

The clinical use of GVS is still little explored in terms of dose, type and mode, intensity, exercise monitoring, and form of application in rehabilitation treatment (18).

This research was submitted to the Research Ethics Committee of the Universidade Metodista de São Paulo according to resolution 466/12, thus, data collection was initiated after its approval. The project was accepted under CAAE number 25693719.8.0000.5508, according to the Declaration of Helsinki.

## Case Description

Patient G, 25 years old, male, single, Brazilian, middle class, height 1.80 m, white, with sequelae of chronic spinal cord trauma was characterized as tetraplegia due to gunshot wound, in continuously attended physician and medical rehabilitation programs. The physical examination prior to the evaluation of the objective instruments selected evidenced muscular spasm and elastic hypertonia in lower limbs as well as hyperreflexia in profound reflexes in lower limbs, poor mobility in upper limbs, and no mobility in lower limbs, with lack of sensory perception in upper and lower limbs and low postural tone in a trunk in the sitting position.

**Figure 1:**
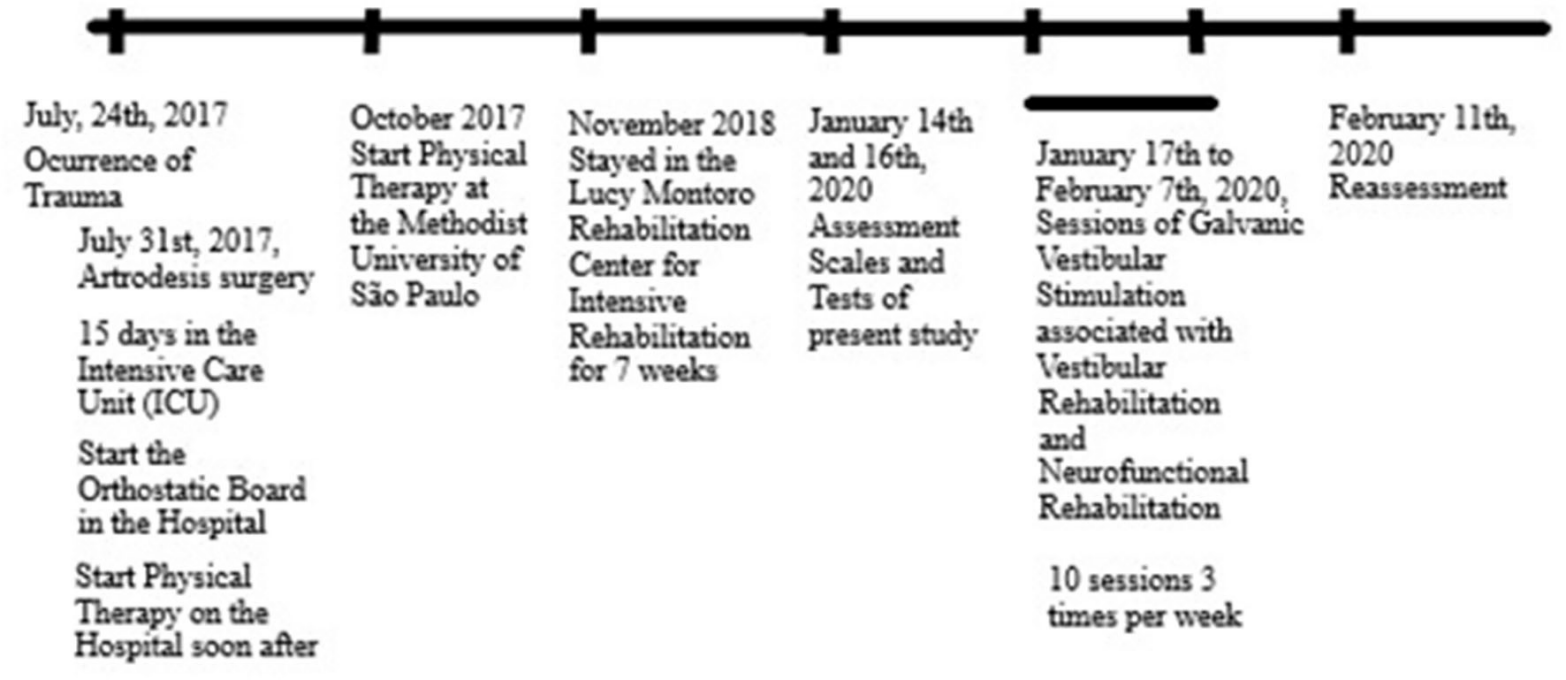
Timeline of the important data of the case care.

The patient reports that his main functional complaint is the change of decubitus from lying down to sitting alone. The patient reports that he continuously uses the medications, Ritimic, Vesicare, and Imipramine. The sequence of events of the trauma is reported in Figure 1.

Considering the time of injury until the present study, the patient was submitted to physical therapy in all the current time with aims as to treat orthostatic hypotension, adapted transfers, locomotion with a wheelchair, daily living activities, as well as strength in upper limbs and postural control emphasizing sensory stimulus that includes vestibular stimulus, with good acceptance and development of performance, but with limitations of postural control, control of wheelchair, and use of upper limbs in reaching, grasping and manipulation.

The participating patient was informed about the study procedures and signed the Informed Consent Form (ICF).

## Diagnostic Assessment, Details on The Therapeutic Intervention, Follow-Up, and Outcomes

The evaluations performed in the present study were the Functional Independence Measure (FIM) (21), the American Spinal Injury Association (ASIA) evaluation (17), and the Clinical Posturography, using force platform to assess postural balance, in the sitting position (with the force platform on a seating bench and the patient been positioned over it), through Sensory Organization and Functional Reach Tests. These are clinical, physiological, and functional tests for the characterization of the independence level, sensory, and motor level of injury, disability grade, and postural control in sitting position, in evidence for trunk control. The medical examination and diagnostic methods were obtained at the moment of the trauma that includes care on tertiary hospitals and segment on secondary rehabilitation centers for spinal cord injury sequel, in this case, tetraplegia.

CT scan evaluation of the thoracic spine on September 15, 2017 showed arthrodesis at C5–T1, metallic artifacts underlying the vertebral body of C6 and C7, posterior column arthrodesis at C5–C7, metallic artifacts within the spinal canal of C6 and C7, disc space reduction at C7–T1, and other structures unchanged.

The pre-treatment FIM showed a total score of 65, showing in self-care the need for assistance and in communication and social cognition the patient's independence. The ASIA evaluation showed motor level C5 on the right and left. The disability grade showed a complete lesion. The type of lesion was tetraplegia.

The pre-treatment evaluation of the sensory organization test of Clinical Posturography through force platform in a sitting position showed very high values for studied variables (Table 1), reflecting poor postural and trunk control instead no explicit movement of the trunk was evidenced.

**Table 1:** The scores of the posturography variables obtained in the evaluation of the sensory organization test in the four test conditions.

	**OE_FP**	**CE_FP**	**OE_MP**	**CE_MP**
	**Filtered**	**Filtered**	**Filtered**	**Filtered**
Total Area (cm^2^)	1499,752737	1499,9908	1500	1500
Slope (Degrees)	12,2902167	7,15542911	165,3378269	134,5571018
Ellipse Area (cm^2^)	1851,746532	2148,335098	2825,726257	2657,640044
Length (cm)	6728,305144	4562,008582	9547,665999	13376,98392
Amplitude X	49,99999984	49,99987186	50	50
Amplitude Y	29,99505483	29,99989288	30	30
X RMS (cm)	15,78072086	17,93648657	18,97090519	16,47124119
Y RMS (cm)	7,049944773	8,595794102	11,03124791	12,16530921
X Variance (cm)	240,0846495	194,7873191	204,4286118	146,07709
Y Variance (cm)	48,32369579	68,21517949	113,4382752	147,3073071
Mean Velocity (cm/s)	122,9498053	71,75677271	40,10040572	34,72367665
% Anterior Mean	0,519770088	0,358573077	0,572657076	0,458679403
% Posterior Mean	0,480229912	0,641426923	0,427342924	0,541320597
% Left Mean	0,592416439	0,81537772	0,779699692	0,74168221
% Right Mean	0,407583561	0,18462228	0,220300308	0,25831779
X Velocity (cm/s)	98,10778662	56,68578878	26,52754007	22,47279954
Y Velocity (cm/s)	52,35135073	30,66078601	23,68666112	21,08684321

*OE_FP, open eyes and fixed platform; CE_FP, closed eyes and fixed platform; OE_MP, open eyes and moving platform; CE_MP, closed eyes and moving platform; X, X-axis or latero-lateral; Y, Y-axis or anteroposterior; RMS, root mean square*.

The most effective information in the pre-treatment sensory evaluation for the spatial variables (ellipse area and total area) was somatosensory. The information that brought more control to the temporal variables (velocity in x and y) was the vestibular information. The sensory evaluation showed a physiological pattern. The functional reach test showed a characteristic, such as a baseline in the sitting position.

The patient was submitted to therapeutic proof after the pre-treatment assessment to have the parameter of the Noise Galvanic Vestibular Stimulation chosen when a drop in a score of posturography evaluation was observed with no sensation of discomfort, showing effect in the postural and trunk control. The selected intensity parameter was 0.7 mA and the main variable for this choice was the decreased velocity of displacement of the center of pressure in this stimulation moment compared to baseline (pre-treatment sensory organization test parameters).

The proposed treatment was 10 (ten) sessions, 3 (three) times per week, of GVS associated with customized vestibular rehabilitation and neurofunctional physical therapy.

Galvanic vestibular stimulation was performed with the parameters of random noise stimulation (RNS) mode, time 40 min, ramp up 20 s, ramp down 20 s, frequency 1 1 (one) Hz, and frequency 2 100 (one hundred) Hz. The stimulation mode is based on the application of alternating current signals with random frequency variation to the patient's circuit (22). The device used was the Microestim TES from NKL ®. The electrodes were 5 × 7 cm, made of silicone with sponge coupling with 0.9% saline solution. The electrodes were placed on the mastoids and secured with an elastic band with Velcro (22). GVS may in the spinal cord injury patient provide stimulation of vestibule and reticulospinal tracts and the pool of lumbosacral neurons and the propriospinal network in sublesional segments improving components of postural balance and mobility as postural tone of trunk control, postural reactions, and mobility that include standard gait generator on the lumbar level. Our aim was centered on postural and trunk control.

Customized vestibular rehabilitation proposed exercises chosen to collaborate and correlate with Noise Galvanic Vestibular Stimulation, such as exercises for vestibular-ocular reflex and vestibulospinal reflex among balance control exercises. A sling was added to vestibular rehabilitation, which is an elastic band 10 cm wide and 2.20-m long, placed on the right lower limb, trunk, and attached to the left shoulder to aid stability, decrease degrees of freedom of movement, resistance, strength, and somatosensory stimulus, and to promote control during the execution of the exercises. The series of exercises was designed to sequentially stimulate head and neck, eye-head and eye-hand coordination, functional reaching exercises with less trunk movement and more stability, trunk movement progression, and anticipatory and compensatory postural adjustment. Stimulating the independence of the patient in all exercises, what was developed during the following days of the session.

The FIM showed changes from the pre- and post-treatment assessments in the self-care part. The total score increased to sixty-eight (68).

The ASIA assessment showed changes between the pre- and post-treatment assessments (Table 2) that include better outcomes levels. One of the outcomes showed the improvement in disability grade from A (complete) to C (incomplete).

**Table 2:** Values of the scores and variables of the American Spinal Injury Association (ASIA) assessment at the initial and final moments of treatment or pre- and post-treatment.

		**Pre**	**Post**
**Motor level**			
	R	C5	C6
	L	C5	C7
**Sensorial level**			
	R pain	T1	C7
	R fine touch	T1	T3
	L pain	T3	C7
	L fine touch	T1	T4
**Motor index**			
	R	5	10
	L	5	15
**Sensorial index**			
	R pain	16	12
	R fine touch	16	20
	R sum	32	32
	L pain	20	12
	L fine touch	16	22
	L sum	36	34
**PPZ**			
	R pain	T3	T1-T2
	R fine touch	T4	NO
	L pain	NO	T1-T2
	L fine touch	T3	NO
	R motor	NO	L2-S1
	L motor	NO	L2-S1
**Degree of disability**			
		A	C

*R, right; L, left; C, cervical spinal cord level and disability grade score; according to scale; T, thoracic spinal cord level; L, lumbar spinal cord level; S, sacral spinal cord level; NO, not observed*.

The post-treatment assessment in Clinical Posturography showed low area, length, amplitude, root mean square (RMS) scores in the X-axis, and velocity relative to the pre-evaluation (Table 3), reflecting an improvement in postural and trunk control in the sensory-motor demand.

**Table 3:** Values of the scores of the variables of the evaluation of the sensory organization test in posturography at the post-treatment or final moment.

	**OE_FP**	**CE_FP**	**OE_MP**	**CE_MP**
	**Filtered**	**Filtered**	**Filtered**	**Filtered**
Total Area (cm^2^)	6,072761965	6,072761965	81,53877231	93,81003507
Slope (Degrees)	176,5226302	170,0284893	121,632587	123,3058214
Ellipse Area (cm^2^)	1,091835585	1,302265738	28,17670975	37,05840091
Length (cm)	127,2021327	225,3931606	323,0843434	406,8310943
Amplitude X	4,229717861	4,229717861	8,372568301	9,518359059
Amplitude Y	1,435736889	1,435736889	9,738800495	9,855694084
X RMS (cm)	3,068585407	3,31624707	2,91730079	2,844704086
Y RMS (cm)	10,38224192	10,47006505	8,681407224	7,585495735
X Variance (cm)	0,164715978	0,196699715	5,87487528	7,760684825
Y Variance (cm)	0,020894539	0,029038634	15,30907374	17,77688299
Mean Velocity (cm/s)	1,999216244	0,786992788	0,76719892	0,79058194
% Anterior Average	0,153961041	0,151043983	0,241670545	0,289802403
% Posterior Average	0,846038959	0,848956017	0,758329455	0,710197597
% Left Average	0,439171439	0,434273432	0,467531066	0,488486197
% Right Average	0,560828561	0,565726568	0,532468934	0,511513803
X Velocity (cm/s)	1,66029533	0,650014533	0,606676892	0,611251723
Y Velocity (cm/s)	0,818697938	0,325706139	0,35235809	0,38032792

*OE_FP, open eyes and fixed platform; CE_FP, closed eyes and fixed platform; OE_MP, open eyes and moving platform; CE_MP, closed eyes and moving platform; X, X-axis or latero-lateral; Y, Y-axis or anteroposterior; RMS, root mean square*.

The sensory analysis observed in the curve of the spatial variable became physiological and more based on somatosensory information, and temporal variables based on visual information (on the x-axis) and somatosensory (on the y-axis), the latter with a physiological curve that reflects a sensory organization of postural and trunk control.

The functional reach test at the post-treatment concerning the basal condition showed an increase in the score of the variables, with a decrease in RMS and mean velocity in the X- and Y-axes. These could be interpreted as an improvement seen in the features of the test to gain amplitude, mobility, and anticipatory postural adjustments to do the excursion to anterior displacement to be stable during postural control displacement of the center of pressure and posture.

## Discussion

The search for recovery from spinal cord injuries is one of the most awaited therapeutics and moves many studies and research studies worldwide (23, 24). Recovery and repair after the incident installing the injury are still far from our resources and tools, however, some researchers have developed instruments that allow functional recovery. Neuromodulation, neurorehabilitation, and brain-machine interface studies have shown that participants showed gains in functional recovery in patients with spinal cord injury with improvement in the leg and arm movement and in their sensitivity (23, 24). The current study showed that it is possible to observe changes in postural control at the level of the lesion, above and below it, promoting functional and physiological effects, and / reduction of pathophysiological effects through the use of GVS in the postural control of a patient with spinal cord injury.

Galvanic vestibular stimulation, in this sense, is a resource that can activate the vestibulospinal and reticulospinal tracts in people without injury that includes the activation of the pool of lumbosacral neurons and the propriospinal network (using for those comfortable currents ranging from 2 to 5 mA), having the potential to activate these structures in people with spinal cord injury at sub-injury levels (18). It is also shown to be capable of being a resource to evaluate the spinal cord through structure and function in patients with spinal cord injury by HTLV-1, using for a GVS with 2 mA (milliamperes) and an electromyography apparatus in gastrocnemius muscle for assessed postural reactions properly to the vestibular system and evaluated the integrity of the spinal cord, vestibular-spinal tracts in the presence of infectious for HTLV-1 to observe functional prognosis on that patients (19).

The clinical pre-evaluation of this case report showed the impairment in the FIM scale (21) paired with the ASIA (17) evaluation since the difficulties and dysfunctions were related to the sensory-motor deficits observed in tetraplegia. The post-treatment evaluation showed an improvement in functionality by the Functional Independence Measurement scale (21) with improvement in self-care with an increase in independence. The outcome was beyond what was expected since the ASIA assessment (17) showed a change in disability grade from A to C or from complete injury to incomplete injury. The classification in this sense post-treatment may have been possible only because, probably, the neural structure of the spinal cord initially did not have a complete lesion and parts of the difficulties were not only structural but also functional. The classification from complete to incomplete means that we had a motor response in all the lumbar and sacral muscle groups, although we did not evaluate the sensitivity of levels S4 and S5 because we did not have the technical structure for this evaluation (10, 17, 20).

The medullary level of the lesion was quite striking since it moved from C5 to C6 and C7 comparing pre- and post-treatment ASIA assessments of this case report. The structural and functional prognosis of a patient with the motor level at C5 and few key muscles is quite limiting, while the functional prognosis of a patient at C7 level, already with key muscles, such as triceps brachii, in addition to the biceps brachii (from C5 motor level), can lead to a functional gain in activities, such as moving the wheelchair, greater ability in posture transfers, especially from lying down to sitting and in the use of the transfer board, in addition to functional activities of daily living, such as eating and dressing, hygiene, among others, since the arm muscles can be activated more forcefully (10, 17, 20). Our patient showed improvement in transfer and postural and trunk control, ranging to reaching, grasping and manipulation, and control of the wheelchair.

The posturographic evaluations showed in the pre-treatment moment that the scores in the baseline condition were high and of probable difficulty for the manifestation of postural control that includes the potential risk of increased pressure and shear forces under the region of the base of support showing low postural and trunk control in the sensory-motor demand.

The pairing of the vestibulo-ocular and vestibulospinal reflexes can be observed in everyday functions with a fine-tuning that allows the image on the retina to remain fixed for a few seconds and postural stability to be maintained in the postural or static moment and the dynamic or progressing moment in different directions and in different functions (25). In addition to hierarchizing and making the vestibular and visual function more compatible with the physiological demand that each pathway receives of information, tuned demand of motricity and postural control. The visual and vestibular function must be of components in the postural control and mobility, for that, we thought not to do the stimulation of GVS alone but to provide the stimulation with sensory demand among physical exercises of vestibular rehabilitation and neurofunctional physical therapy to provide the training of this sensory reflexes, reactions, and postural and mobility developments.

Noise Galvanic Vestibular Stimulation has been studied with the concept of stochastic resonance (26), which indicates that in a non-linear system, such as the vestibular system, a low residual function can be amplified when imperceptible noise is added. The striking characteristic of stochastic resonance is a bell-shaped curved response to an increase in the strength of the noise; implying that stimulation at very low or very high amplitudes will degrade the evoked response and that amplitude between these upper and lower limits will result in improved outcomes, even in healthy subjects with this addition leading to a lower threshold for evoking the vestibulospinal reflex in posture in these individuals. This effect was seen for stimulation amplitudes ranging from 0.3 and 1.1 mA (26). The trunk and postural control must be improved in this context even in the healthy subjects reflecting postural tone and reactions based on the vestibular system and their connections. We expected if this could be possible in the spinal cord injury patient who has a healthy vestibular system but the poor trunk and postural control due to sensory-motor sequel.

To this characteristic, we observed our patient's response to the therapeutic proof performed. The therapeutic proof allowed us to know the electrical current within the parameters of functionality with the maximum control response observed, especially in the sway velocity variable, and the sensory curve observed was more supported on the vestibular stimulus. The therapeutic proof has already been used in a study with elderly people, identifying the parameter for each subject capable of optimizing postural function (the mean stimulation intensity of the optimal stimulus was 178.8 (±9.1) μA) (9). The phenomenon of stochastic resonance may help to understand that each subject responds individually to the vestibular stimulus under the phenomenon, and thus the identification of this parameter is important for the stimulus to be perhaps more assertive for the effect of controlling postural sway and ocular stability (9, 26). This helped us to fine tune the assertive stimulation to our patients during the following sessions compounding to the physical exercises providing for his own response to the stimulation.

A temporary positive effect of Noise Galvanic Vestibular Stimulation has been reported in one study, even without stimulation being active at the acute time, and the effect on balance improvement remained for a few hours even after stimulation ceased. Furthermore, it has been suggested that combining vestibular rehabilitation exercises with GVS may be beneficial for overall results, as we observed in this patient (26). The effect of the stimulation observed in the therapeutic proof improving to the 10 sessions of stimulation plus physical sensory-motor exercises gave an outcome with development of trunk and postural control that include not only temporal variables but spatial variables of postural control also.

The feasibility of chronic motion modulation with stimulation, however, remains to be defined (26). The current study showed the effect of GVS for some time is equal to the treatment plan and each stimulation session, since it proposed a standardized stimulation time of ten (10) sessions with intervals, three (3) per week, to compose a frequency of physiological and functional stimulation. The effect on postural control showed a great decrease in the scores of postural variables that includes area, displacement, and velocity, with modulation of the RMS indicating a lower risk of falling in this patient partially or gradually, as well as an augmentation in postural control parameters in the sitting posture, some of these results were more expressive than the observed on therapeutic proof, in one moment.

The vestibular and reticular systems make a parallel and almost independent system of the corticospinal system, although these two systems act together for everyday functions. The improvement in postural control also indicates that there is direct and indirect connectivity of the vestibular and reticular system with other brainstem nuclei and cortical and subcortical areas (13, 16).

The postural evaluation of the sensory organization test and the functional reach test in sitting position on the force platform allowed the investigation of trunk control changes in postural control and the correlation with the scales, its repercussions on the individual's functionality, and the response systems. The postural evaluation of these tests in the force platform brings a new perspective on postural evaluations of postural control showed a way to evaluate the reference posture of patients who use the sitting posture in everyday life and functionality, especially patients who use the wheelchair most of the time, or who have limitations for standing posture and gait. The importance of this evaluation helps our study and others that may come to verify treatment, similar evaluations, and therapies.

The main limitations of our study are in the fact that it is a single clinical case report with fewer factors for analysis and strength and consistency of quantitative data in terms of evidence. A limitation of the actual study was the clinical assessment of muscle tone, muscular spasms and hypertonia have only prior initial parameters, and not final parameters also, in part because the instruments of the assessment make the evaluation in pre- and post-moments large in time. Posturographic assessment in the sitting posture seems to be poorly described in the literature and we found no studies to allow us to discuss the data with the same model of assessment we used it. GVS has many studies (26, 27) with its use to trigger vestibular function, and a few studies (9, 27) with a clinical focus, and criteria for frequency of sessions and therapy time, as well as therapeutic dose parameters, are still poorly known. We observed characteristics of a period close to 1 month of therapy and its outcomes, and follow-up observations and new designs are needed to observe open questions.

## Patient Perspective

Patient report about the study and therapy proposed: “In December 2019, I was invited by Professor C. and her student T., to participate in a study with electrostimulation. At first, I was a little afraid, because when anyone hears that they are going to receive stimulation through electricity, they already create a certain fear in their head, but in the very first sessions, the two of them gave me a lot of confidence and we began the study. In the first sessions that I received the electrostimulation, I felt a slight discomfort in the stimulated area, but soon afterward I began to adapt and noticed the positive results. Being more specific about my impressions of the sessions, I can say that it is extremely tranquil, painless and that it was very positive for my recovery. I usually don't have much insight into the improvements I have during any kind of therapy, but during the study sessions, I soon realized that I was managing to do some movements that I had a lot of difficulty in a much easier way.

I have no doubt that participating in this study has made me believe that soon other types of therapies may be used for patients with the same diagnosis as mine, and I hope that each one of them will achieve further improvements.

I would like to thank Professor C., my friend and physiotherapist T., and the Methodist University and its professionals.”

## Final Considerations

This is a case report that proposed Noise Galvanic Vestibular Stimulation associated with vestibular rehabilitation and neurofunctional physical therapy in the spinal cord injury patient with objective outcomes denoting improvement of the trunk and postural control through physical, physiological, and functional assessment and that could be perceived and appropriated by the patient as the development of the impairment of the sequel of tetraplegia. Studies with other patients and a bigger number of patients must be conducted to seek the corroborating of these results.

## Data Availability Statement

The original contributions presented in the study are included in the article/supplementary material, further inquiries can be directed to the corresponding author/s.

## Ethics Statement

This research was submitted to the Research Ethics Committee of the Universidade Metodista de São Paulo according to resolution 466/12, thus, data collection was initiated after its approval. The project was accepted under CAAE number 25693719.8.0000.5508, according to the Declaration of Helsinki. The patients/participants provided their written informed consent to participate in this study.

## Author Contributions

All authors listed have made a substantial, direct, and intellectual contribution to the work and approved it for publication.

## Conflict of Interest

The authors declare that the research was conducted in the absence of any commercial or financial relationships that could be construed as a potential conflict of interest.

## Publisher's Note

All claims expressed in this article are solely those of the authors and do not necessarily represent those of their affiliated organizations, or those of the publisher, the editors and the reviewers. Any product that may be evaluated in this article, or claim that may be made by its manufacturer, is not guaranteed or endorsed by the publisher.
